# (1*R*,2*S*)-Methyl 1-(4-chloro­phen­yl)-3-oxo-1,2,3,4-tetra­hydro­cyclo­penta­[*b*]indole-2-carboxyl­ate 0.2-hydrate

**DOI:** 10.1107/S1600536811042358

**Published:** 2011-10-22

**Authors:** Sadiya Raja, Michael Bolte

**Affiliations:** a Institute of Organic Chemistry, RWTH Aachen University, Landoltweg 1, 52074 Aachen, Germany; b Institut für Anorganische Chemie, J. W. Goethe-Universität Frankfurt, Max-von-Laue-Strasse 7, 60438 Frankfurt/Main, Germany

## Abstract

The title compound, C_19_H_14_ClNO_3_·0.2H_2_O, crystallizes with five mol­ecules and a disordered water mol­ecule in the asymmetric unit. Four of the five mol­ecules form hydrogen-bonded dimers *via* N—H⋯O hydrogen bonds towards another symmetry-independent mol­ecule, whereas the fifth mol­ecule forms a hydrogen-bonded dimer with its symmetry equivalent, also *via* N—H⋯O hydrogen bonds. The dihedral angle between the planes of the fused benzene ring and the five-membered ring to which it is attached is 79.45 (13), 49.00 (15), 72.49 (16), 81.91 (18) and 76.38 (16)° for the five mol­ecules in the asymmetric unit.

## Related literature

For biological and pharmaceutical properties of cyclo­pent[*b*]indole, see: Bergman & Venemalm (1990 ▶). For the synthesis of cyclo­pentenones, see: Gibson *et al.* (2004 ▶); for the Naza­rov cyclization, see: Shimada *et al.* (2011 ▶); Vaidya *et al.* (2011 ▶); for the synthetic procedure, see Rueping & Raja (2011 ▶).
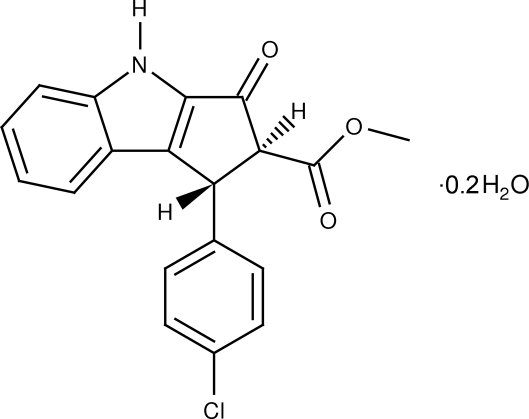


## Experimental

### 

#### Crystal data

C_19_H_14_ClNO_3_·0.2H_2_O
*M* *_r_* = 343.37
Monoclinic, 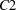

*a* = 73.342 (4) Å
*b* = 9.6065 (4) Å
*c* = 11.8737 (7) Å
β = 92.432 (4)°
*V* = 8358.2 (8) Å^3^
*Z* = 20
Mo *K*α radiation
μ = 0.25 mm^−1^
*T* = 173 K
0.42 × 0.42 × 0.22 mm

#### Data collection

Stoe IPDS II two-circle diffractometer
Absorption correction: multi-scan (*MULABS*; Spek, 2009 ▶; Blessing, 1995 ▶) *T*_min_ = 0.904, *T*_max_ = 0.948
29836 measured reflections
14845 independent reflections
9911 reflections with *I* > 2σ(*I*)
*R*_int_ = 0.057

#### Refinement

*R*[*F* ^2^ > 2σ(*F* ^2^)] = 0.055
*wR*(*F* ^2^) = 0.144
*S* = 0.90
14845 reflections
1107 parameters
1 restraint
H atoms treated by a mixture of independent and constrained refinement
Δρ_max_ = 1.21 e Å^−3^
Δρ_min_ = −0.35 e Å^−3^
Absolute structure: Flack (1983 ▶), 6346 Friedel pairs
Flack parameter: −0.01 (5)

### 

Data collection: *X-AREA* (Stoe & Cie, 2001 ▶); cell refinement: *X-AREA*; data reduction: *X-AREA*; program(s) used to solve structure: *SHELXS97* (Sheldrick, 2008 ▶); program(s) used to refine structure: *SHELXL97* (Sheldrick, 2008 ▶); molecular graphics: *XP* (Sheldrick, 2008 ▶); software used to prepare material for publication: *SHELXL97*.

## Supplementary Material

Crystal structure: contains datablock(s) I, global. DOI: 10.1107/S1600536811042358/im2327sup1.cif

Structure factors: contains datablock(s) I. DOI: 10.1107/S1600536811042358/im2327Isup2.hkl

Supplementary material file. DOI: 10.1107/S1600536811042358/im2327Isup3.cml

Additional supplementary materials:  crystallographic information; 3D view; checkCIF report

## Figures and Tables

**Table 1:** Hydrogen-bond geometry (Å, °)

*D*—H⋯*A*	*D*—H	H⋯*A*	*D*⋯*A*	*D*—H⋯*A*
N1—H1⋯O1^i^	0.76 (5)	2.24 (5)	2.827 (4)	134 (5)
N1*A*—H1*A*⋯O1*B*	0.86 (5)	2.13 (5)	2.992 (5)	172 (4)
N1*B*—H1*B*⋯O1*A*	0.90 (6)	1.90 (6)	2.767 (5)	161 (5)
N1*C*—H1*C*⋯O1*D*	1.04 (7)	1.97 (7)	2.966 (5)	160 (6)
N1*D*—H1*D*⋯O1*C*	0.95 (5)	1.89 (5)	2.806 (5)	159 (4)

Symmetry code: (i) 
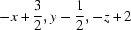
.

**Fig. 1.**
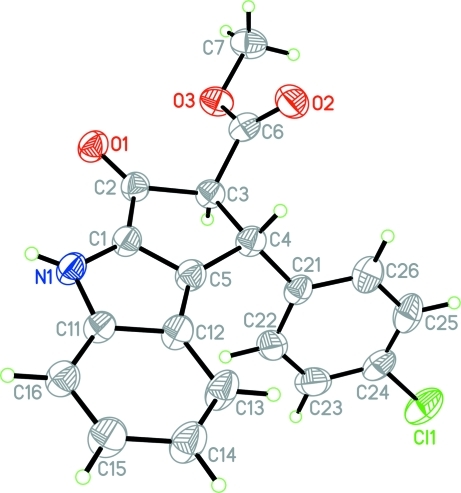
Perspective view of one molecule in the asymmetric unit of the title compound with the atom numbering; displacement ellipsoids are depicted at the 50% probability level.

## supplementary crystallographic information

### Comment

The structural unit of cyclopentenones is present in many natural products. Especially the cyclopent[*b*]indole core is a common moiety in alkaloids with interesting biological and pharmaceutical properties (Bergman & Venemalm, 1990). Due to the great importance of cyclopentenones in organic chemistry different methods have been developed for their synthesis (Gibson *et al.*, 2004). One of the most versatile methods is the Nazarov cyclization, which can be catalyzed by Brønsted or Lewis acids (Shimada *et al.*, 2011; Vaidya *et al.*, 2011). The title compound was prepared for the first time using this methodology in the presence of a chiral metal complex.

The title compound crystallizes with five molecules and a disordered water molecule in the asymmetric unit. Four of the five molecules form hydrogen bonded dimers *via* N—H···O hydrogen bonds towards another symmetry independent molecule, whereas the fifth molecule forms a hydrogen bonded dimer with its symmetry equivalent, also *via* N—H···O hydrogen bonds. The dihedral angle between the planes of the phenyl ring and the five-membered ring to which it is attached is 79.45 (13)°, 49.00 (15)°, 72.49 (16)°, 81.91 (18)°, 76.38 (16)°, repectively, for the five molecules in the asymmetric unit.

### Experimental

The title compound has been synthesized as described by Rueping & Raja (2011).

### Refinement

All H atoms bonded to C and N could be located by difference Fourier synthesis. Those bonded to C were refined with fixed individual displacement parameters [U(H) = 1.2 *U*_eq_(C) or U(H) = 1.5 *U*_eq_(C_methyl_)] using a riding model with C—H ranging from 0.95Å to 1.00 Å. H atoms bonded to N were freely refined. The H atoms of the disordered water molecules could not be located and were omitted from refinement. The carbonyl O atom in one molecule is disordered over two positions with a site occupation factor of 0.50 (1) for each occupied site. In another molecule the complete acetato residue is disordered over two positions with a site occupation factor of 0.613 (6) for the major occupied site. The disordered atoms (O2C/O2C' and O2D, O3D, C7D/O2D', O3D', C7D') and the water molecules were only isotropically refined.

### Crystal data

**Table:**

C_19_H_14_ClNO_3_·0.2H_2_O	*F*(000) = 3560
*M**_r_* = 343.37	*D*_x_ = 1.364 Mg m^−^^3^
Monoclinic, *C*2	Mo *K*α radiation, λ = 0.71073 Å
Hall symbol: C 2y	Cell parameters from 19373 reflections
*a* = 73.342 (4) Å	θ = 1.8–27.8°
*b* = 9.6065 (4) Å	µ = 0.25 mm^−^^1^
*c* = 11.8737 (7) Å	*T* = 173 K
β = 92.432 (4)°	Plate, colourless
*V* = 8358.2 (8) Å^3^	0.42 × 0.42 × 0.22 mm
*Z* = 20	

### Data collection

**Table:**

Stoe IPDS II two-circle diffractometer	14845 independent reflections
Radiation source: fine-focus sealed tube	9911 reflections with *I* > 2σ(*I*)
graphite	*R*_int_ = 0.057
ω scans	θ_max_ = 26.4°, θ_min_ = 1.7°
Absorption correction: multi-scan (*MULABS*; Spek, 2009; Blessing, 1995)	*h* = −91→91
*T*_min_ = 0.904, *T*_max_ = 0.948	*k* = −10→12
29836 measured reflections	*l* = −14→14

### Refinement

**Table:**

Refinement on *F*^2^	Secondary atom site location: difference Fourier map
Least-squares matrix: full	Hydrogen site location: inferred from neighbouring sites
*R*[*F*^2^ > 2σ(*F*^2^)] = 0.055	H atoms treated by a mixture of independent and constrained refinement
*wR*(*F*^2^) = 0.144	*w* = 1/[σ^2^(*F*_o_^2^) + (0.087*P*)^2^] where *P* = (*F*_o_^2^ + 2*F*_c_^2^)/3
*S* = 0.90	(Δ/σ)_max_ = 0.001
14845 reflections	Δρ_max_ = 1.21 e Å^−^^3^
1107 parameters	Δρ_min_ = −0.35 e Å^−^^3^
1 restraint	Absolute structure: Flack (1983), 6346 Friedel pairs
Primary atom site location: structure-invariant direct methods	Flack parameter: −0.01 (5)

### Special details

**Table:**

Experimental. ;
Geometry. All e.s.d.'s (except the e.s.d. in the dihedral angle between two l.s. planes) are estimated using the full covariance matrix. The cell e.s.d.'s are taken into account individually in the estimation of e.s.d.'s in distances, angles and torsion angles; correlations between e.s.d.'s in cell parameters are only used when they are defined by crystal symmetry. An approximate (isotropic) treatment of cell e.s.d.'s is used for estimating e.s.d.'s involving l.s. planes.
Refinement. Refinement of *F*^2^ against ALL reflections. The weighted *R*-factor *wR* and goodness of fit *S* are based on *F*^2^, conventional *R*-factors *R* are based on *F*, with *F* set to zero for negative *F*^2^. The threshold expression of *F*^2^ > σ(*F*^2^) is used only for calculating *R*-factors(gt) *etc*. and is not relevant to the choice of reflections for refinement. *R*-factors based on *F*^2^ are statistically about twice as large as those based on *F*, and *R*- factors based on ALL data will be even larger.

### Fractional atomic coordinates and isotropic or equivalent isotropic displacement parameters (Å^2^)

**Table:**

	*x*	*y*	*z*	*U*_iso_*/*U*_eq_	Occ. (<1)
Cl1	0.777880 (18)	1.08585 (12)	0.31634 (8)	0.0577 (3)	
N1	0.76018 (5)	0.4903 (3)	0.8737 (2)	0.0352 (7)	
H1	0.7542 (6)	0.475 (5)	0.923 (4)	0.052 (13)*	
O1	0.75795 (4)	0.7794 (3)	1.00830 (18)	0.0389 (6)	
O2	0.80263 (4)	0.9687 (3)	0.9110 (2)	0.0474 (7)	
O3	0.77559 (4)	1.0536 (3)	0.96284 (19)	0.0391 (6)	
C1	0.76626 (5)	0.6214 (4)	0.8604 (3)	0.0332 (8)	
C2	0.76519 (5)	0.7518 (4)	0.9201 (3)	0.0324 (8)	
C3	0.77462 (5)	0.8586 (4)	0.8444 (3)	0.0326 (8)	
H3	0.7647	0.9113	0.8024	0.039*	
C4	0.78499 (5)	0.7750 (4)	0.7562 (3)	0.0339 (8)	
H4	0.7981	0.7682	0.7817	0.041*	
C5	0.77626 (5)	0.6341 (4)	0.7666 (3)	0.0349 (8)	
C6	0.78620 (5)	0.9645 (4)	0.9090 (3)	0.0338 (8)	
C7	0.78512 (6)	1.1707 (4)	1.0187 (3)	0.0478 (10)	
H7A	0.7762	1.2301	1.0550	0.072*	
H7B	0.7915	1.2251	0.9626	0.072*	
H7C	0.7940	1.1351	1.0756	0.072*	
C11	0.76605 (5)	0.4129 (4)	0.7839 (3)	0.0323 (8)	
C12	0.77622 (5)	0.5021 (4)	0.7143 (3)	0.0366 (8)	
C13	0.78373 (6)	0.4464 (4)	0.6159 (3)	0.0454 (10)	
H13	0.7906	0.5031	0.5679	0.055*	
C14	0.78078 (7)	0.3096 (4)	0.5921 (3)	0.0513 (11)	
H14	0.7856	0.2715	0.5259	0.062*	
C15	0.77079 (6)	0.2225 (4)	0.6622 (3)	0.0466 (10)	
H15	0.7692	0.1272	0.6428	0.056*	
C16	0.76329 (5)	0.2727 (4)	0.7580 (3)	0.0376 (8)	
H16	0.7565	0.2141	0.8049	0.045*	
C21	0.78368 (5)	0.8420 (4)	0.6408 (3)	0.0324 (8)	
C22	0.76766 (6)	0.8339 (4)	0.5730 (3)	0.0457 (10)	
H22	0.7578	0.7783	0.5962	0.055*	
C23	0.76610 (7)	0.9068 (5)	0.4720 (3)	0.0510 (11)	
H23	0.7552	0.9026	0.4264	0.061*	
C24	0.78071 (6)	0.9853 (4)	0.4391 (3)	0.0421 (9)	
C25	0.79677 (6)	0.9934 (4)	0.5028 (3)	0.0468 (10)	
H25	0.8067	1.0471	0.4781	0.056*	
C26	0.79813 (6)	0.9211 (4)	0.6041 (3)	0.0433 (9)	
H26	0.8091	0.9259	0.6491	0.052*	
Cl1A	0.778617 (17)	0.47059 (13)	0.19660 (11)	0.0656 (3)	
N1A	0.88405 (5)	0.9286 (4)	0.0884 (3)	0.0492 (9)	
H1A	0.8946 (6)	0.957 (5)	0.114 (3)	0.047 (12)*	
O1A	0.86839 (4)	1.1049 (4)	0.2920 (3)	0.0645 (9)	
O2A	0.82536 (4)	1.2061 (3)	0.1614 (2)	0.0505 (7)	
O3A	0.82890 (4)	1.1645 (3)	0.3469 (2)	0.0483 (7)	
C1A	0.86729 (5)	0.9484 (4)	0.1336 (3)	0.0415 (9)	
C2A	0.86058 (6)	1.0277 (4)	0.2248 (3)	0.0428 (9)	
C3A	0.84014 (5)	0.9953 (4)	0.2256 (3)	0.0379 (8)	
H3A	0.8378	0.9358	0.2924	0.045*	
C4A	0.83517 (5)	0.9113 (4)	0.1156 (3)	0.0352 (8)	
H4A	0.8294	0.9777	0.0600	0.042*	
C5A	0.85405 (5)	0.8772 (4)	0.0754 (3)	0.0377 (9)	
C6A	0.83038 (5)	1.1312 (4)	0.2372 (3)	0.0378 (8)	
C7A	0.82215 (8)	1.3052 (5)	0.3685 (4)	0.0639 (13)	
H7A1	0.8216	1.3200	0.4499	0.096*	
H7A2	0.8099	1.3161	0.3332	0.096*	
H7A3	0.8304	1.3738	0.3367	0.096*	
C11A	0.88126 (6)	0.8382 (5)	−0.0012 (3)	0.0463 (10)	
C12A	0.86248 (5)	0.8026 (5)	−0.0130 (3)	0.0432 (9)	
C13A	0.85655 (7)	0.7111 (5)	−0.0978 (3)	0.0540 (11)	
H13A	0.8440	0.6874	−0.1083	0.065*	
C14A	0.86950 (8)	0.6561 (7)	−0.1659 (4)	0.0752 (16)	
H14A	0.8658	0.5903	−0.2221	0.090*	
C15A	0.88753 (8)	0.6938 (7)	−0.1549 (4)	0.0801 (18)	
H15A	0.8958	0.6562	−0.2062	0.096*	
C16A	0.89408 (7)	0.7832 (6)	−0.0731 (4)	0.0614 (13)	
H16A	0.9067	0.8067	−0.0655	0.074*	
C21A	0.82174 (5)	0.7973 (4)	0.1342 (3)	0.0350 (8)	
C22A	0.80448 (5)	0.8318 (4)	0.1702 (3)	0.0401 (9)	
H22A	0.8017	0.9272	0.1817	0.048*	
C23A	0.79130 (6)	0.7344 (4)	0.1897 (3)	0.0432 (9)	
H23A	0.7797	0.7614	0.2156	0.052*	
C24A	0.79531 (6)	0.5950 (4)	0.1705 (3)	0.0437 (9)	
C25A	0.81193 (6)	0.5553 (4)	0.1337 (3)	0.0456 (10)	
H25A	0.8144	0.4599	0.1198	0.055*	
C26A	0.82524 (6)	0.6569 (4)	0.1169 (3)	0.0410 (9)	
H26A	0.8369	0.6293	0.0931	0.049*	
N1B	0.90201 (5)	1.2311 (4)	0.3303 (3)	0.0537 (9)	
H1B	0.8914 (8)	1.195 (6)	0.302 (4)	0.074 (16)*	
O1B	0.92143 (4)	1.0344 (3)	0.1526 (3)	0.0659 (9)	
O2B	0.95768 (6)	1.2788 (4)	0.0891 (3)	0.0833 (12)	
O3B	0.96435 (4)	1.0542 (3)	0.0887 (2)	0.0538 (7)	
C1B	0.91969 (6)	1.2042 (4)	0.3019 (4)	0.0518 (11)	
C2B	0.92818 (6)	1.1128 (5)	0.2241 (4)	0.0545 (11)	
C3B	0.94887 (6)	1.1371 (4)	0.2446 (3)	0.0466 (10)	
H3B	0.9545	1.0511	0.2781	0.056*	
C4B	0.95127 (6)	1.2585 (4)	0.3302 (3)	0.0434 (9)	
H4B	0.9559	1.3417	0.2896	0.052*	
C5B	0.93183 (5)	1.2862 (4)	0.3603 (3)	0.0418 (9)	
C6B	0.95728 (6)	1.1670 (5)	0.1333 (3)	0.0503 (11)	
C7B	0.97263 (8)	1.0741 (6)	−0.0187 (4)	0.0711 (14)	
H7B1	0.9773	0.9849	−0.0452	0.107*	
H7B2	0.9827	1.1409	−0.0099	0.107*	
H7B3	0.9635	1.1099	−0.0738	0.107*	
C11B	0.90304 (6)	1.3354 (4)	0.4104 (3)	0.0448 (10)	
C12B	0.92160 (6)	1.3739 (5)	0.4304 (3)	0.0459 (10)	
C13B	0.92558 (7)	1.4816 (6)	0.5074 (3)	0.0587 (12)	
H13B	0.9379	1.5094	0.5232	0.070*	
C14B	0.91154 (7)	1.5464 (6)	0.5597 (4)	0.0685 (14)	
H14B	0.9142	1.6200	0.6112	0.082*	
C15B	0.89346 (7)	1.5061 (6)	0.5385 (4)	0.0678 (15)	
H15B	0.8841	1.5518	0.5771	0.081*	
C16B	0.88889 (6)	1.4019 (5)	0.4632 (3)	0.0537 (11)	
H16B	0.8765	1.3762	0.4479	0.064*	
C21B	0.96396 (5)	1.2310 (5)	0.4328 (3)	0.0474 (10)	
C22B	0.96689 (6)	1.0992 (6)	0.4764 (4)	0.0590 (12)	
H22B	0.9616	1.0206	0.4388	0.071*	
C23B	0.97765 (7)	1.0811 (7)	0.5769 (4)	0.0764 (18)	
H23B	0.9795	0.9905	0.6075	0.092*	
C24B	0.98539 (7)	1.1926 (8)	0.6296 (4)	0.082 (2)	
C25B	0.98265 (7)	1.3242 (7)	0.5887 (4)	0.0721 (16)	
H25B	0.9880	1.4019	0.6270	0.087*	
C26B	0.97181 (6)	1.3435 (6)	0.4886 (4)	0.0597 (13)	
H26B	0.9699	1.4347	0.4595	0.072*	
Cl1C	0.82053 (2)	0.45513 (16)	0.83487 (13)	0.0846 (4)	
N1C	0.90607 (6)	−0.0407 (5)	0.5280 (3)	0.0695 (13)	
H1C	0.9147 (9)	−0.058 (7)	0.462 (5)	0.11 (2)*	
O1C	0.87321 (6)	−0.2006 (5)	0.3944 (3)	0.0963 (15)	
O2C	0.84869 (12)	−0.3142 (8)	0.5978 (6)	0.067 (3)*	0.501 (13)
O2C'	0.83800 (11)	−0.2786 (7)	0.6282 (5)	0.057 (3)*	0.499 (13)
O3C	0.83186 (4)	−0.2142 (3)	0.4544 (2)	0.0550 (8)	
C1C	0.88786 (7)	−0.0633 (5)	0.5407 (3)	0.0605 (13)	
C2C	0.87327 (8)	−0.1290 (6)	0.4795 (3)	0.0659 (14)	
C3C	0.85587 (7)	−0.0885 (4)	0.5405 (3)	0.0510 (11)	
H3C	0.8496	−0.0120	0.4968	0.061*	
C4C	0.86239 (7)	−0.0297 (5)	0.6573 (3)	0.0538 (11)	
H4C	0.8618	−0.1062	0.7141	0.065*	
C5C	0.88204 (7)	−0.0008 (5)	0.6375 (3)	0.0541 (11)	
C6C	0.84317 (9)	−0.2103 (5)	0.5433 (4)	0.0690 (15)	
C7C	0.81948 (9)	−0.3322 (5)	0.4441 (4)	0.0735 (15)	
H7C1	0.8118	−0.3225	0.3751	0.110*	
H7C2	0.8118	−0.3349	0.5095	0.110*	
H7C3	0.8266	−0.4185	0.4407	0.110*	
C11C	0.91246 (7)	0.0403 (5)	0.6177 (3)	0.0561 (12)	
C12C	0.89772 (8)	0.0641 (5)	0.6892 (4)	0.0646 (13)	
C13C	0.90121 (12)	0.1458 (11)	0.7844 (7)	0.160 (5)	
H13C	0.8916	0.1701	0.8318	0.192*	
C14C	0.91889 (12)	0.1915 (12)	0.8096 (9)	0.189 (6)	
H14C	0.9216	0.2361	0.8797	0.227*	
C15C	0.93262 (10)	0.1736 (8)	0.7355 (7)	0.109 (3)	
H15C	0.9442	0.2162	0.7501	0.130*	
C16C	0.92958 (8)	0.0932 (6)	0.6394 (4)	0.0670 (14)	
H16C	0.9391	0.0754	0.5899	0.080*	
C21C	0.85189 (7)	0.0921 (5)	0.6991 (3)	0.0562 (12)	
C22C	0.84164 (8)	0.0793 (6)	0.7934 (4)	0.0655 (13)	
H22C	0.8409	−0.0083	0.8301	0.079*	
C23C	0.83253 (8)	0.1926 (6)	0.8349 (5)	0.0758 (16)	
H23C	0.8260	0.1831	0.9020	0.091*	
C24C	0.83269 (7)	0.3165 (6)	0.7818 (4)	0.0654 (13)	
C25C	0.84252 (9)	0.3329 (6)	0.6879 (5)	0.0797 (17)	
H25C	0.8427	0.4203	0.6506	0.096*	
C26C	0.85224 (9)	0.2212 (5)	0.6473 (4)	0.0759 (16)	
H26C	0.8593	0.2335	0.5828	0.091*	
Cl1B	0.99885 (2)	1.1688 (3)	0.75259 (12)	0.1279 (9)	
Cl1D	0.95500 (2)	−0.26305 (14)	−0.36464 (8)	0.0733 (4)	
N1D	0.89851 (6)	−0.3381 (4)	0.2628 (3)	0.0513 (9)	
H1D	0.8915 (6)	−0.303 (5)	0.322 (4)	0.053 (12)*	
O1D	0.93180 (5)	−0.1525 (4)	0.3645 (2)	0.0656 (9)	
O2D	0.96722 (15)	−0.3306 (12)	0.3683 (9)	0.084 (3)*	0.388 (6)
O3D	0.98058 (13)	−0.2325 (10)	0.2264 (7)	0.071 (3)*	0.388 (6)
O2D'	0.96920 (8)	−0.4203 (7)	0.2918 (5)	0.0735 (18)*	0.612 (6)
O3D'	0.97143 (8)	−0.1960 (6)	0.3186 (4)	0.0645 (17)*	0.612 (6)
C1D	0.91638 (7)	−0.3158 (4)	0.2384 (3)	0.0475 (10)	
C2D	0.93127 (6)	−0.2334 (4)	0.2846 (3)	0.0509 (11)	
C3D	0.94734 (6)	−0.2609 (5)	0.2076 (3)	0.0485 (10)	
H3D	0.9496	−0.1750	0.1627	0.058*	
C4D	0.94105 (7)	−0.3806 (4)	0.1252 (3)	0.0504 (11)	
H4D	0.9478	−0.4675	0.1472	0.060*	
C5D	0.92141 (7)	−0.3984 (5)	0.1528 (3)	0.0551 (12)	
C6D	0.96417 (7)	−0.2959 (5)	0.2760 (3)	0.0578 (12)	
C7D	0.9985 (2)	−0.2562 (18)	0.2820 (12)	0.080 (4)*	0.388 (6)
H7D1	1.0019	−0.1758	0.3293	0.120*	0.388 (6)
H7D2	1.0076	−0.2686	0.2248	0.120*	0.388 (6)
H7D3	0.9981	−0.3399	0.3289	0.120*	0.388 (6)
C7D'	0.98801 (13)	−0.2213 (11)	0.3876 (7)	0.073 (2)*	0.612 (6)
H7D4	0.9965	−0.1437	0.3791	0.109*	0.612 (6)
H7D5	0.9937	−0.3079	0.3633	0.109*	0.612 (6)
H7D6	0.9849	−0.2295	0.4669	0.109*	0.612 (6)
C11D	0.89184 (7)	−0.4388 (5)	0.1911 (3)	0.0544 (11)	
C12D	0.90581 (8)	−0.4826 (5)	0.1210 (3)	0.0598 (13)	
C13D	0.90185 (12)	−0.5863 (7)	0.0421 (5)	0.114 (3)	
H13D	0.9109	−0.6174	−0.0072	0.137*	
C14D	0.88484 (13)	−0.6423 (9)	0.0367 (6)	0.133 (4)	
H14D	0.8822	−0.7139	−0.0167	0.159*	
C15D	0.87153 (11)	−0.5998 (6)	0.1050 (5)	0.090 (2)	
H15D	0.8599	−0.6427	0.0977	0.108*	
C16D	0.87430 (8)	−0.4967 (5)	0.1844 (4)	0.0606 (13)	
H16D	0.8649	−0.4668	0.2316	0.073*	
C21D	0.94443 (6)	−0.3485 (4)	0.0008 (3)	0.0474 (10)	
C22D	0.94143 (6)	−0.2190 (5)	−0.0450 (3)	0.0461 (10)	
H22D	0.9371	−0.1464	0.0013	0.055*	
C23D	0.94456 (6)	−0.1914 (5)	−0.1575 (3)	0.0531 (11)	
H23D	0.9427	−0.1006	−0.1876	0.064*	
C24D	0.95036 (7)	−0.2970 (5)	−0.2238 (3)	0.0527 (11)	
C25D	0.95285 (9)	−0.4295 (6)	−0.1824 (4)	0.0731 (15)	
H25D	0.9566	−0.5028	−0.2301	0.088*	
C26D	0.94986 (9)	−0.4539 (5)	−0.0699 (4)	0.0727 (16)	
H26D	0.9516	−0.5451	−0.0404	0.087*	
O1W	0.00055 (6)	0.4508 (5)	−0.0639 (3)	0.0315 (10)*	0.50
O2W	0.00588 (6)	0.7119 (5)	−0.0531 (3)	0.0304 (10)*	0.50

### Atomic displacement parameters (Å^2^)

**Table:**

	*U*^11^	*U*^22^	*U*^33^	*U*^12^	*U*^13^	*U*^23^
Cl1	0.0888 (9)	0.0489 (6)	0.0365 (4)	0.0117 (6)	0.0137 (5)	0.0092 (4)
N1	0.046 (2)	0.0317 (17)	0.0286 (14)	−0.0005 (15)	0.0101 (13)	−0.0016 (12)
O1	0.0477 (16)	0.0365 (14)	0.0332 (12)	−0.0016 (12)	0.0095 (11)	−0.0039 (10)
O2	0.0387 (17)	0.0534 (18)	0.0505 (14)	−0.0056 (14)	0.0040 (11)	−0.0097 (13)
O3	0.0434 (16)	0.0312 (14)	0.0426 (13)	0.0021 (12)	−0.0003 (11)	−0.0090 (11)
C1	0.035 (2)	0.033 (2)	0.0316 (16)	−0.0006 (16)	0.0042 (14)	−0.0001 (13)
C2	0.0305 (19)	0.034 (2)	0.0327 (16)	0.0001 (16)	0.0025 (14)	0.0006 (14)
C3	0.035 (2)	0.0295 (19)	0.0335 (16)	0.0009 (16)	0.0024 (14)	−0.0014 (14)
C4	0.039 (2)	0.0291 (19)	0.0336 (16)	0.0007 (16)	0.0055 (14)	−0.0027 (14)
C5	0.041 (2)	0.032 (2)	0.0327 (16)	−0.0004 (17)	0.0048 (15)	−0.0001 (14)
C6	0.042 (2)	0.0307 (19)	0.0293 (15)	0.0055 (17)	0.0020 (14)	0.0005 (14)
C7	0.059 (3)	0.035 (2)	0.049 (2)	−0.002 (2)	−0.0060 (19)	−0.0093 (18)
C11	0.039 (2)	0.0271 (18)	0.0307 (15)	0.0032 (16)	0.0038 (14)	0.0000 (13)
C12	0.050 (2)	0.0230 (19)	0.0378 (17)	0.0015 (16)	0.0143 (16)	−0.0012 (14)
C13	0.067 (3)	0.027 (2)	0.0442 (19)	0.0016 (19)	0.0274 (18)	−0.0042 (15)
C14	0.075 (3)	0.033 (2)	0.047 (2)	−0.001 (2)	0.024 (2)	−0.0046 (17)
C15	0.066 (3)	0.028 (2)	0.047 (2)	0.002 (2)	0.0136 (18)	−0.0031 (16)
C16	0.046 (2)	0.032 (2)	0.0352 (17)	0.0024 (17)	0.0063 (15)	0.0027 (14)
C21	0.043 (2)	0.0248 (18)	0.0303 (15)	0.0012 (16)	0.0098 (14)	−0.0030 (13)
C22	0.060 (3)	0.040 (2)	0.0371 (18)	−0.018 (2)	0.0013 (17)	0.0026 (16)
C23	0.070 (3)	0.046 (2)	0.0356 (19)	−0.010 (2)	−0.0047 (18)	−0.0023 (17)
C24	0.060 (3)	0.036 (2)	0.0309 (16)	0.009 (2)	0.0106 (17)	−0.0017 (15)
C25	0.056 (3)	0.038 (2)	0.047 (2)	0.004 (2)	0.0192 (18)	0.0111 (17)
C26	0.045 (2)	0.041 (2)	0.0441 (19)	0.0072 (19)	0.0083 (17)	0.0065 (16)
Cl1A	0.0576 (7)	0.0454 (6)	0.0949 (8)	−0.0177 (6)	0.0138 (6)	−0.0090 (6)
N1A	0.036 (2)	0.060 (2)	0.0510 (19)	−0.0020 (18)	−0.0038 (16)	−0.0005 (16)
O1A	0.0442 (18)	0.069 (2)	0.080 (2)	−0.0033 (16)	−0.0071 (15)	−0.0297 (18)
O2A	0.059 (2)	0.0400 (17)	0.0518 (15)	0.0035 (14)	−0.0064 (13)	0.0019 (13)
O3A	0.0615 (19)	0.0381 (16)	0.0458 (14)	−0.0012 (14)	0.0093 (12)	−0.0033 (12)
C1A	0.034 (2)	0.044 (2)	0.0460 (19)	−0.0029 (18)	−0.0028 (16)	0.0022 (17)
C2A	0.038 (2)	0.040 (2)	0.050 (2)	−0.0031 (18)	−0.0061 (17)	−0.0008 (17)
C3A	0.040 (2)	0.037 (2)	0.0371 (17)	0.0002 (17)	0.0004 (15)	0.0006 (15)
C4A	0.039 (2)	0.0292 (19)	0.0377 (17)	0.0014 (16)	0.0003 (15)	0.0004 (14)
C5A	0.040 (2)	0.037 (2)	0.0369 (17)	−0.0017 (17)	−0.0002 (15)	0.0078 (15)
C6A	0.038 (2)	0.030 (2)	0.045 (2)	−0.0063 (17)	0.0017 (16)	−0.0008 (16)
C7A	0.084 (4)	0.039 (3)	0.070 (3)	0.005 (2)	0.014 (2)	−0.015 (2)
C11A	0.045 (2)	0.057 (3)	0.0372 (18)	0.002 (2)	0.0023 (16)	0.0075 (18)
C12A	0.042 (2)	0.048 (2)	0.0402 (19)	−0.0013 (19)	0.0031 (16)	0.0075 (17)
C13A	0.054 (3)	0.062 (3)	0.047 (2)	0.001 (2)	0.0033 (19)	−0.007 (2)
C14A	0.068 (4)	0.099 (4)	0.058 (3)	0.005 (3)	0.001 (2)	−0.024 (3)
C15A	0.070 (4)	0.116 (5)	0.054 (3)	0.015 (4)	0.010 (2)	−0.012 (3)
C16A	0.048 (3)	0.083 (4)	0.053 (2)	0.009 (3)	0.008 (2)	−0.001 (2)
C21A	0.034 (2)	0.035 (2)	0.0358 (17)	0.0001 (16)	−0.0018 (14)	−0.0030 (14)
C22A	0.037 (2)	0.031 (2)	0.052 (2)	0.0020 (17)	−0.0031 (17)	0.0002 (16)
C23A	0.034 (2)	0.041 (2)	0.054 (2)	−0.0016 (19)	0.0029 (16)	−0.0055 (18)
C24A	0.041 (2)	0.035 (2)	0.055 (2)	−0.0085 (18)	0.0025 (17)	−0.0042 (17)
C25A	0.054 (3)	0.033 (2)	0.050 (2)	−0.0030 (19)	0.0049 (18)	−0.0059 (17)
C26A	0.040 (2)	0.034 (2)	0.049 (2)	−0.0003 (18)	0.0050 (16)	−0.0035 (16)
N1B	0.037 (2)	0.044 (2)	0.079 (2)	0.0002 (18)	−0.0107 (18)	−0.0074 (18)
O1B	0.052 (2)	0.051 (2)	0.093 (2)	−0.0003 (16)	−0.0156 (17)	−0.0278 (17)
O2B	0.134 (4)	0.046 (2)	0.070 (2)	0.011 (2)	−0.001 (2)	0.0160 (17)
O3B	0.0577 (19)	0.0491 (18)	0.0542 (15)	−0.0036 (15)	−0.0017 (13)	0.0005 (13)
C1B	0.042 (3)	0.038 (2)	0.075 (3)	−0.001 (2)	−0.013 (2)	−0.004 (2)
C2B	0.050 (3)	0.034 (2)	0.079 (3)	−0.001 (2)	−0.013 (2)	−0.004 (2)
C3B	0.039 (2)	0.035 (2)	0.065 (2)	−0.0031 (18)	−0.0115 (18)	0.0059 (18)
C4B	0.043 (2)	0.039 (2)	0.048 (2)	−0.0029 (18)	−0.0093 (17)	0.0029 (16)
C5B	0.039 (2)	0.035 (2)	0.050 (2)	−0.0026 (18)	−0.0106 (17)	0.0052 (17)
C6B	0.048 (3)	0.042 (3)	0.060 (2)	0.006 (2)	−0.0159 (19)	0.001 (2)
C7B	0.080 (4)	0.079 (4)	0.055 (2)	−0.016 (3)	0.008 (2)	−0.001 (2)
C11B	0.047 (3)	0.038 (2)	0.048 (2)	−0.001 (2)	−0.0060 (18)	0.0068 (17)
C12B	0.045 (2)	0.048 (2)	0.044 (2)	−0.002 (2)	−0.0055 (17)	0.0066 (17)
C13B	0.052 (3)	0.072 (3)	0.052 (2)	−0.013 (3)	0.0006 (19)	−0.010 (2)
C14B	0.057 (3)	0.089 (4)	0.060 (3)	−0.009 (3)	0.007 (2)	−0.028 (3)
C15B	0.063 (3)	0.093 (4)	0.048 (2)	0.000 (3)	0.011 (2)	−0.010 (2)
C16B	0.047 (3)	0.065 (3)	0.049 (2)	−0.005 (2)	0.0033 (19)	0.008 (2)
C21B	0.033 (2)	0.058 (3)	0.051 (2)	−0.006 (2)	−0.0053 (17)	0.0105 (19)
C22B	0.042 (3)	0.071 (3)	0.063 (3)	−0.002 (2)	−0.004 (2)	0.020 (2)
C23B	0.037 (3)	0.113 (5)	0.078 (3)	0.005 (3)	−0.004 (2)	0.048 (3)
C24B	0.034 (3)	0.149 (7)	0.064 (3)	−0.017 (3)	−0.008 (2)	0.043 (4)
C25B	0.052 (3)	0.111 (5)	0.052 (2)	−0.030 (3)	−0.012 (2)	0.005 (3)
C26B	0.052 (3)	0.073 (3)	0.053 (2)	−0.019 (3)	−0.012 (2)	0.012 (2)
Cl1C	0.0721 (9)	0.0727 (9)	0.1102 (10)	−0.0061 (8)	0.0179 (8)	−0.0413 (8)
N1C	0.068 (3)	0.097 (3)	0.0435 (19)	0.037 (3)	0.0005 (18)	−0.011 (2)
O1C	0.094 (3)	0.129 (4)	0.064 (2)	0.057 (3)	−0.0185 (19)	−0.049 (2)
O3C	0.071 (2)	0.0443 (18)	0.0503 (16)	0.0021 (16)	0.0030 (14)	0.0047 (13)
C1C	0.073 (4)	0.066 (3)	0.043 (2)	0.031 (3)	0.005 (2)	−0.003 (2)
C2C	0.081 (4)	0.076 (4)	0.041 (2)	0.033 (3)	−0.002 (2)	−0.003 (2)
C3C	0.074 (3)	0.039 (2)	0.041 (2)	0.007 (2)	0.0023 (19)	0.0028 (17)
C4C	0.077 (3)	0.042 (2)	0.044 (2)	−0.007 (2)	0.016 (2)	−0.0010 (18)
C5C	0.074 (3)	0.046 (3)	0.044 (2)	0.000 (2)	0.017 (2)	0.0008 (18)
C6C	0.122 (5)	0.041 (3)	0.043 (2)	−0.008 (3)	−0.008 (2)	0.0119 (19)
C7C	0.117 (5)	0.044 (3)	0.060 (3)	−0.009 (3)	0.005 (3)	−0.003 (2)
C11C	0.066 (3)	0.054 (3)	0.050 (2)	0.009 (2)	0.017 (2)	0.010 (2)
C12C	0.082 (4)	0.050 (3)	0.064 (3)	−0.010 (3)	0.032 (2)	−0.014 (2)
C13C	0.115 (6)	0.219 (11)	0.152 (7)	−0.082 (7)	0.085 (5)	−0.138 (7)
C14C	0.115 (7)	0.232 (12)	0.229 (10)	−0.097 (7)	0.093 (7)	−0.186 (10)
C15C	0.078 (4)	0.091 (5)	0.161 (6)	−0.033 (4)	0.052 (4)	−0.045 (5)
C16C	0.075 (4)	0.058 (3)	0.070 (3)	0.010 (3)	0.025 (3)	0.020 (3)
C21C	0.071 (3)	0.048 (3)	0.052 (2)	−0.014 (2)	0.022 (2)	−0.011 (2)
C22C	0.088 (4)	0.049 (3)	0.062 (3)	−0.013 (3)	0.030 (2)	−0.006 (2)
C23C	0.076 (4)	0.073 (4)	0.081 (3)	−0.022 (3)	0.041 (3)	−0.010 (3)
C24C	0.064 (3)	0.059 (3)	0.074 (3)	−0.015 (3)	0.019 (2)	−0.027 (3)
C25C	0.118 (5)	0.040 (3)	0.084 (3)	−0.004 (3)	0.033 (3)	0.001 (2)
C26C	0.112 (5)	0.047 (3)	0.072 (3)	−0.004 (3)	0.052 (3)	−0.001 (2)
Cl1B	0.0537 (8)	0.260 (3)	0.0683 (8)	−0.0291 (12)	−0.0207 (7)	0.0696 (12)
Cl1D	0.1082 (11)	0.0733 (8)	0.0391 (5)	−0.0386 (8)	0.0109 (5)	−0.0019 (5)
N1D	0.062 (3)	0.045 (2)	0.0473 (18)	0.0042 (19)	0.0088 (17)	−0.0058 (16)
O1D	0.068 (2)	0.076 (2)	0.0528 (16)	0.0070 (19)	0.0031 (15)	−0.0278 (16)
C1D	0.067 (3)	0.035 (2)	0.0407 (19)	0.008 (2)	0.0045 (19)	−0.0006 (16)
C2D	0.064 (3)	0.041 (2)	0.047 (2)	0.011 (2)	−0.0001 (19)	−0.0043 (18)
C3D	0.067 (3)	0.039 (2)	0.0401 (19)	0.008 (2)	0.0057 (18)	0.0001 (16)
C4D	0.073 (3)	0.039 (2)	0.041 (2)	0.001 (2)	0.0145 (19)	0.0022 (16)
C5D	0.088 (4)	0.038 (2)	0.040 (2)	0.001 (2)	0.017 (2)	0.0044 (17)
C6D	0.076 (3)	0.052 (3)	0.046 (2)	0.010 (3)	0.000 (2)	0.0003 (19)
C11D	0.080 (3)	0.043 (3)	0.040 (2)	0.000 (2)	0.005 (2)	0.0015 (18)
C12D	0.088 (4)	0.043 (3)	0.049 (2)	−0.020 (2)	0.017 (2)	−0.0094 (19)
C13D	0.163 (7)	0.094 (5)	0.092 (4)	−0.074 (5)	0.076 (4)	−0.057 (4)
C14D	0.172 (8)	0.129 (7)	0.102 (5)	−0.091 (6)	0.067 (5)	−0.071 (5)
C15D	0.123 (6)	0.072 (4)	0.078 (4)	−0.050 (4)	0.017 (3)	−0.014 (3)
C16D	0.081 (4)	0.046 (3)	0.055 (2)	−0.012 (3)	0.004 (2)	0.009 (2)
C21D	0.066 (3)	0.037 (2)	0.041 (2)	−0.003 (2)	0.0143 (18)	−0.0032 (17)
C22D	0.049 (3)	0.047 (2)	0.0429 (19)	−0.001 (2)	0.0041 (17)	0.0008 (17)
C23D	0.062 (3)	0.056 (3)	0.041 (2)	−0.010 (2)	−0.0047 (18)	0.0068 (19)
C24D	0.068 (3)	0.050 (3)	0.0401 (19)	−0.022 (2)	0.0027 (18)	−0.0006 (18)
C25D	0.115 (5)	0.051 (3)	0.055 (2)	−0.004 (3)	0.028 (3)	−0.011 (2)
C26D	0.130 (5)	0.040 (3)	0.050 (2)	0.000 (3)	0.026 (3)	0.002 (2)

### Geometric parameters (Å, °)

**Table:**

Cl1—C24	1.754 (4)	C14B—C15B	1.394 (7)
N1—C1	1.347 (5)	C14B—H14B	0.9500
N1—C11	1.384 (5)	C15B—C16B	1.375 (7)
N1—H1	0.76 (5)	C15B—H15B	0.9500
O1—C2	1.223 (4)	C16B—H16B	0.9500
O2—C6	1.205 (4)	C21B—C26B	1.380 (7)
O3—C6	1.337 (4)	C21B—C22B	1.381 (7)
O3—C7	1.468 (5)	C22B—C23B	1.413 (6)
C1—C5	1.365 (5)	C22B—H22B	0.9500
C1—C2	1.443 (5)	C23B—C24B	1.353 (9)
C2—C3	1.546 (5)	C23B—H23B	0.9500
C3—C6	1.513 (5)	C24B—C25B	1.367 (9)
C3—C4	1.546 (5)	C24B—Cl1B	1.742 (5)
C3—H3	1.0000	C25B—C26B	1.413 (6)
C4—C5	1.504 (5)	C25B—H25B	0.9500
C4—C21	1.514 (5)	C26B—H26B	0.9500
C4—H4	1.0000	Cl1C—C24C	1.736 (5)
C5—C12	1.412 (5)	N1C—C1C	1.368 (7)
C7—H7A	0.9800	N1C—C11C	1.384 (6)
C7—H7B	0.9800	N1C—H1C	1.04 (7)
C7—H7C	0.9800	O1C—C2C	1.222 (5)
C11—C16	1.394 (5)	O2C—C6C	1.248 (8)
C11—C12	1.424 (5)	O2C'—C6C	1.275 (8)
C12—C13	1.418 (5)	O3C—C6C	1.315 (5)
C13—C14	1.359 (6)	O3C—C7C	1.453 (6)
C13—H13	0.9500	C1C—C5C	1.380 (6)
C14—C15	1.408 (6)	C1C—C2C	1.415 (8)
C14—H14	0.9500	C2C—C3C	1.543 (7)
C15—C16	1.372 (5)	C3C—C6C	1.497 (7)
C15—H15	0.9500	C3C—C4C	1.554 (6)
C16—H16	0.9500	C3C—H3C	1.0000
C21—C26	1.389 (6)	C4C—C5C	1.496 (7)
C21—C22	1.398 (5)	C4C—C21C	1.496 (7)
C22—C23	1.389 (5)	C4C—H4C	1.0000
C22—H22	0.9500	C5C—C12C	1.424 (7)
C23—C24	1.381 (6)	C7C—H7C1	0.9800
C23—H23	0.9500	C7C—H7C2	0.9800
C24—C25	1.374 (6)	C7C—H7C3	0.9800
C25—C26	1.389 (5)	C11C—C16C	1.368 (7)
C25—H25	0.9500	C11C—C12C	1.421 (7)
C26—H26	0.9500	C12C—C13C	1.391 (8)
Cl1A—C24A	1.748 (4)	C13C—C14C	1.390 (11)
N1A—C1A	1.375 (5)	C13C—H13C	0.9500
N1A—C11A	1.382 (6)	C14C—C15C	1.376 (9)
N1A—H1A	0.86 (5)	C14C—H14C	0.9500
O1A—C2A	1.215 (5)	C15C—C16C	1.388 (8)
O2A—C6A	1.197 (4)	C15C—H15C	0.9500
O3A—C6A	1.350 (4)	C16C—H16C	0.9500
O3A—C7A	1.467 (5)	C21C—C22C	1.381 (6)
C1A—C5A	1.353 (5)	C21C—C26C	1.385 (7)
C1A—C2A	1.429 (6)	C22C—C23C	1.379 (8)
C2A—C3A	1.531 (6)	C22C—H22C	0.9500
C3A—C6A	1.498 (5)	C23C—C24C	1.347 (8)
C3A—C4A	1.565 (5)	C23C—H23C	0.9500
C3A—H3A	1.0000	C24C—C25C	1.362 (7)
C4A—C21A	1.496 (5)	C25C—C26C	1.386 (8)
C4A—C5A	1.519 (6)	C25C—H25C	0.9500
C4A—H4A	1.0000	C26C—H26C	0.9500
C5A—C12A	1.433 (6)	Cl1D—C24D	1.751 (4)
C7A—H7A1	0.9800	N1D—C11D	1.366 (6)
C7A—H7A2	0.9800	N1D—C1D	1.372 (6)
C7A—H7A3	0.9800	N1D—H1D	0.95 (5)
C11A—C16A	1.400 (6)	O1D—C2D	1.227 (5)
C11A—C12A	1.421 (6)	O2D—C6D	1.158 (11)
C12A—C13A	1.392 (6)	O3D—C7D	1.465 (17)
C13A—C14A	1.378 (7)	O3D—C6D	1.492 (11)
C13A—H13A	0.9500	O2D'—C6D	1.262 (8)
C14A—C15A	1.372 (8)	O3D'—C6D	1.199 (7)
C14A—H14A	0.9500	O3D'—C7D'	1.457 (10)
C15A—C16A	1.368 (8)	C1D—C5D	1.352 (6)
C15A—H15A	0.9500	C1D—C2D	1.438 (6)
C16A—H16A	0.9500	C2D—C3D	1.544 (6)
C21A—C26A	1.390 (5)	C3D—C6D	1.487 (6)
C21A—C22A	1.393 (5)	C3D—C4D	1.567 (6)
C22A—C23A	1.372 (6)	C3D—H3D	1.0000
C22A—H22A	0.9500	C4D—C5D	1.500 (7)
C23A—C24A	1.392 (6)	C4D—C21D	1.540 (5)
C23A—H23A	0.9500	C4D—H4D	1.0000
C24A—C25A	1.366 (6)	C5D—C12D	1.438 (7)
C25A—C26A	1.401 (6)	C7D—H7D1	0.9800
C25A—H25A	0.9500	C7D—H7D2	0.9800
C26A—H26A	0.9500	C7D—H7D3	0.9800
N1B—C1B	1.378 (6)	C7D'—H7D4	0.9800
N1B—C11B	1.381 (6)	C7D'—H7D5	0.9800
N1B—H1B	0.90 (6)	C7D'—H7D6	0.9800
O1B—C2B	1.223 (5)	C11D—C16D	1.400 (7)
O2B—C6B	1.197 (5)	C11D—C12D	1.411 (7)
O3B—C6B	1.322 (5)	C12D—C13D	1.391 (7)
O3B—C7B	1.448 (5)	C13D—C14D	1.358 (10)
C1B—C5B	1.357 (6)	C13D—H13D	0.9500
C1B—C2B	1.435 (7)	C14D—C15D	1.359 (10)
C2B—C3B	1.544 (6)	C14D—H14D	0.9500
C3B—C6B	1.509 (6)	C15D—C16D	1.376 (7)
C3B—C4B	1.552 (6)	C15D—H15D	0.9500
C3B—H3B	1.0000	C16D—H16D	0.9500
C4B—C5B	1.509 (6)	C21D—C22D	1.372 (6)
C4B—C21B	1.525 (5)	C21D—C26D	1.384 (6)
C4B—H4B	1.0000	C22D—C23D	1.390 (5)
C5B—C12B	1.420 (6)	C22D—H22D	0.9500
C7B—H7B1	0.9800	C23D—C24D	1.363 (7)
C7B—H7B2	0.9800	C23D—H23D	0.9500
C7B—H7B3	0.9800	C24D—C25D	1.374 (7)
C11B—C16B	1.391 (6)	C25D—C26D	1.383 (6)
C11B—C12B	1.421 (6)	C25D—H25D	0.9500
C12B—C13B	1.403 (6)	C26D—H26D	0.9500
C13B—C14B	1.374 (7)	O1W—O1W^i^	1.522 (8)
C13B—H13B	0.9500	O2W—O2W^i^	1.557 (9)
			
C1—N1—C11	107.3 (3)	C13B—C14B—H14B	119.4
C1—N1—H1	119 (4)	C15B—C14B—H14B	119.4
C11—N1—H1	134 (4)	C16B—C15B—C14B	121.6 (5)
C6—O3—C7	115.5 (3)	C16B—C15B—H15B	119.2
N1—C1—C5	111.8 (3)	C14B—C15B—H15B	119.2
N1—C1—C2	136.8 (3)	C15B—C16B—C11B	117.5 (4)
C5—C1—C2	111.4 (3)	C15B—C16B—H16B	121.2
O1—C2—C1	130.0 (3)	C11B—C16B—H16B	121.2
O1—C2—C3	125.1 (3)	C26B—C21B—C22B	118.9 (4)
C1—C2—C3	104.8 (3)	C26B—C21B—C4B	118.4 (4)
C6—C3—C2	114.0 (3)	C22B—C21B—C4B	122.5 (4)
C6—C3—C4	114.3 (3)	C21B—C22B—C23B	120.1 (5)
C2—C3—C4	107.1 (3)	C21B—C22B—H22B	120.0
C6—C3—H3	107.0	C23B—C22B—H22B	120.0
C2—C3—H3	107.0	C24B—C23B—C22B	120.1 (5)
C4—C3—H3	107.0	C24B—C23B—H23B	119.9
C5—C4—C21	116.5 (3)	C22B—C23B—H23B	119.9
C5—C4—C3	100.9 (3)	C23B—C24B—C25B	121.0 (4)
C21—C4—C3	112.2 (3)	C23B—C24B—Cl1B	119.7 (5)
C5—C4—H4	108.9	C25B—C24B—Cl1B	119.3 (5)
C21—C4—H4	108.9	C24B—C25B—C26B	119.3 (5)
C3—C4—H4	108.9	C24B—C25B—H25B	120.3
C1—C5—C12	106.7 (3)	C26B—C25B—H25B	120.3
C1—C5—C4	113.1 (3)	C21B—C26B—C25B	120.6 (5)
C12—C5—C4	139.9 (3)	C21B—C26B—H26B	119.7
O2—C6—O3	124.9 (3)	C25B—C26B—H26B	119.7
O2—C6—C3	124.8 (3)	C1C—N1C—C11C	107.8 (4)
O3—C6—C3	110.3 (3)	C1C—N1C—H1C	133 (4)
O3—C7—H7A	109.5	C11C—N1C—H1C	118 (4)
O3—C7—H7B	109.5	C6C—O3C—C7C	117.4 (4)
H7A—C7—H7B	109.5	N1C—C1C—C5C	111.1 (4)
O3—C7—H7C	109.5	N1C—C1C—C2C	137.3 (4)
H7A—C7—H7C	109.5	C5C—C1C—C2C	111.6 (5)
H7B—C7—H7C	109.5	O1C—C2C—C1C	130.6 (5)
N1—C11—C16	130.0 (3)	O1C—C2C—C3C	123.8 (5)
N1—C11—C12	108.1 (3)	C1C—C2C—C3C	105.6 (4)
C16—C11—C12	121.8 (3)	C6C—C3C—C2C	109.9 (4)
C5—C12—C13	135.1 (3)	C6C—C3C—C4C	115.7 (3)
C5—C12—C11	106.0 (3)	C2C—C3C—C4C	106.3 (4)
C13—C12—C11	118.8 (3)	C6C—C3C—H3C	108.2
C14—C13—C12	118.2 (4)	C2C—C3C—H3C	108.2
C14—C13—H13	120.9	C4C—C3C—H3C	108.2
C12—C13—H13	120.9	C5C—C4C—C21C	114.8 (4)
C13—C14—C15	122.3 (4)	C5C—C4C—C3C	100.9 (3)
C13—C14—H14	118.9	C21C—C4C—C3C	115.9 (4)
C15—C14—H14	118.9	C5C—C4C—H4C	108.3
C16—C15—C14	121.2 (4)	C21C—C4C—H4C	108.3
C16—C15—H15	119.4	C3C—C4C—H4C	108.3
C14—C15—H15	119.4	C1C—C5C—C12C	106.1 (4)
C15—C16—C11	117.6 (3)	C1C—C5C—C4C	112.6 (4)
C15—C16—H16	121.2	C12C—C5C—C4C	141.1 (4)
C11—C16—H16	121.2	O2C—C6C—O2C'	43.8 (4)
C26—C21—C22	119.0 (3)	O2C—C6C—O3C	125.0 (5)
C26—C21—C4	119.8 (3)	O2C'—C6C—O3C	114.9 (6)
C22—C21—C4	121.1 (3)	O2C—C6C—C3C	116.6 (5)
C23—C22—C21	120.3 (4)	O2C'—C6C—C3C	128.8 (5)
C23—C22—H22	119.8	O3C—C6C—C3C	112.2 (4)
C21—C22—H22	119.8	O3C—C7C—H7C1	109.5
C24—C23—C22	118.9 (4)	O3C—C7C—H7C2	109.5
C24—C23—H23	120.5	H7C1—C7C—H7C2	109.5
C22—C23—H23	120.5	O3C—C7C—H7C3	109.5
C25—C24—C23	122.2 (3)	H7C1—C7C—H7C3	109.5
C25—C24—Cl1	119.7 (3)	H7C2—C7C—H7C3	109.5
C23—C24—Cl1	118.1 (3)	C16C—C11C—N1C	129.2 (4)
C24—C25—C26	118.5 (4)	C16C—C11C—C12C	122.9 (5)
C24—C25—H25	120.8	N1C—C11C—C12C	108.0 (5)
C26—C25—H25	120.8	C13C—C12C—C11C	117.2 (5)
C25—C26—C21	121.1 (4)	C13C—C12C—C5C	135.6 (5)
C25—C26—H26	119.4	C11C—C12C—C5C	107.0 (4)
C21—C26—H26	119.4	C12C—C13C—C14C	119.4 (6)
C1A—N1A—C11A	106.4 (4)	C12C—C13C—H13C	120.3
C1A—N1A—H1A	129 (3)	C14C—C13C—H13C	120.3
C11A—N1A—H1A	125 (3)	C15C—C14C—C13C	121.6 (7)
C6A—O3A—C7A	115.4 (3)	C15C—C14C—H14C	119.2
C5A—C1A—N1A	111.4 (4)	C13C—C14C—H14C	119.2
C5A—C1A—C2A	113.2 (4)	C16C—C15C—C14C	119.9 (6)
N1A—C1A—C2A	135.4 (4)	C16C—C15C—H15C	120.0
O1A—C2A—C1A	130.9 (4)	C14C—C15C—H15C	120.0
O1A—C2A—C3A	123.7 (4)	C11C—C16C—C15C	118.4 (5)
C1A—C2A—C3A	105.4 (3)	C11C—C16C—H16C	120.8
C6A—C3A—C2A	107.2 (3)	C15C—C16C—H16C	120.8
C6A—C3A—C4A	115.5 (3)	C22C—C21C—C26C	117.4 (5)
C2A—C3A—C4A	107.0 (3)	C22C—C21C—C4C	120.3 (4)
C6A—C3A—H3A	109.0	C26C—C21C—C4C	122.3 (4)
C2A—C3A—H3A	109.0	C23C—C22C—C21C	120.5 (5)
C4A—C3A—H3A	109.0	C23C—C22C—H22C	119.7
C21A—C4A—C5A	120.2 (3)	C21C—C22C—H22C	119.7
C21A—C4A—C3A	112.7 (3)	C24C—C23C—C22C	121.1 (5)
C5A—C4A—C3A	101.0 (3)	C24C—C23C—H23C	119.4
C21A—C4A—H4A	107.4	C22C—C23C—H23C	119.4
C5A—C4A—H4A	107.4	C23C—C24C—C25C	120.0 (5)
C3A—C4A—H4A	107.4	C23C—C24C—Cl1C	119.6 (4)
C1A—C5A—C12A	107.7 (4)	C25C—C24C—Cl1C	120.4 (5)
C1A—C5A—C4A	112.0 (3)	C24C—C25C—C26C	119.6 (5)
C12A—C5A—C4A	139.9 (3)	C24C—C25C—H25C	120.2
O2A—C6A—O3A	123.2 (4)	C26C—C25C—H25C	120.2
O2A—C6A—C3A	126.0 (3)	C21C—C26C—C25C	121.3 (4)
O3A—C6A—C3A	110.7 (3)	C21C—C26C—H26C	119.4
O3A—C7A—H7A1	109.5	C25C—C26C—H26C	119.4
O3A—C7A—H7A2	109.5	C11D—N1D—C1D	107.4 (4)
H7A1—C7A—H7A2	109.5	C11D—N1D—H1D	121 (3)
O3A—C7A—H7A3	109.5	C1D—N1D—H1D	131 (3)
H7A1—C7A—H7A3	109.5	C7D—O3D—C6D	118.9 (9)
H7A2—C7A—H7A3	109.5	C6D—O3D'—C7D'	116.7 (6)
N1A—C11A—C16A	128.7 (4)	C5D—C1D—N1D	111.2 (4)
N1A—C11A—C12A	109.8 (4)	C5D—C1D—C2D	112.5 (4)
C16A—C11A—C12A	121.5 (4)	N1D—C1D—C2D	136.2 (4)
C13A—C12A—C11A	119.7 (4)	O1D—C2D—C1D	130.0 (4)
C13A—C12A—C5A	135.6 (4)	O1D—C2D—C3D	124.5 (4)
C11A—C12A—C5A	104.7 (3)	C1D—C2D—C3D	105.5 (3)
C14A—C13A—C12A	117.7 (5)	C6D—C3D—C2D	110.6 (3)
C14A—C13A—H13A	121.2	C6D—C3D—C4D	113.0 (4)
C12A—C13A—H13A	121.2	C2D—C3D—C4D	106.4 (4)
C15A—C14A—C13A	121.9 (5)	C6D—C3D—H3D	108.9
C15A—C14A—H14A	119.1	C2D—C3D—H3D	108.9
C13A—C14A—H14A	119.1	C4D—C3D—H3D	108.9
C16A—C15A—C14A	122.8 (5)	C5D—C4D—C21D	115.3 (4)
C16A—C15A—H15A	118.6	C5D—C4D—C3D	101.9 (3)
C14A—C15A—H15A	118.6	C21D—C4D—C3D	113.4 (3)
C15A—C16A—C11A	116.4 (5)	C5D—C4D—H4D	108.6
C15A—C16A—H16A	121.8	C21D—C4D—H4D	108.6
C11A—C16A—H16A	121.8	C3D—C4D—H4D	108.6
C26A—C21A—C22A	116.9 (4)	C1D—C5D—C12D	106.7 (4)
C26A—C21A—C4A	124.2 (3)	C1D—C5D—C4D	113.1 (4)
C22A—C21A—C4A	118.9 (3)	C12D—C5D—C4D	140.0 (4)
C23A—C22A—C21A	123.1 (4)	O2D—C6D—O3D'	76.5 (6)
C23A—C22A—H22A	118.5	O2D—C6D—O2D'	62.9 (6)
C21A—C22A—H22A	118.5	O3D'—C6D—O2D'	125.0 (5)
C22A—C23A—C24A	118.2 (4)	O2D—C6D—C3D	134.8 (7)
C22A—C23A—H23A	120.9	O3D'—C6D—C3D	113.1 (5)
C24A—C23A—H23A	120.9	O2D'—C6D—C3D	121.7 (5)
C25A—C24A—C23A	121.2 (4)	O2D—C6D—O3D	111.4 (7)
C25A—C24A—Cl1A	120.4 (3)	O3D'—C6D—O3D	59.3 (5)
C23A—C24A—Cl1A	118.3 (3)	O2D'—C6D—O3D	102.1 (6)
C24A—C25A—C26A	119.2 (4)	C3D—C6D—O3D	110.9 (5)
C24A—C25A—H25A	120.4	O3D—C7D—H7D1	109.5
C26A—C25A—H25A	120.4	O3D—C7D—H7D2	109.5
C21A—C26A—C25A	121.4 (4)	H7D1—C7D—H7D2	109.5
C21A—C26A—H26A	119.3	O3D—C7D—H7D3	109.5
C25A—C26A—H26A	119.3	H7D1—C7D—H7D3	109.5
C1B—N1B—C11B	106.3 (4)	H7D2—C7D—H7D3	109.5
C1B—N1B—H1B	130 (3)	O3D'—C7D'—H7D4	109.5
C11B—N1B—H1B	124 (3)	O3D'—C7D'—H7D5	109.5
C6B—O3B—C7B	115.6 (4)	H7D4—C7D'—H7D5	109.5
C5B—C1B—N1B	111.7 (4)	O3D'—C7D'—H7D6	109.5
C5B—C1B—C2B	113.0 (4)	H7D4—C7D'—H7D6	109.5
N1B—C1B—C2B	135.3 (4)	H7D5—C7D'—H7D6	109.5
O1B—C2B—C1B	130.4 (4)	N1D—C11D—C16D	128.4 (4)
O1B—C2B—C3B	124.7 (4)	N1D—C11D—C12D	109.2 (4)
C1B—C2B—C3B	104.9 (4)	C16D—C11D—C12D	122.4 (4)
C6B—C3B—C2B	109.0 (3)	C13D—C12D—C11D	118.4 (5)
C6B—C3B—C4B	113.0 (3)	C13D—C12D—C5D	136.1 (5)
C2B—C3B—C4B	107.5 (4)	C11D—C12D—C5D	105.5 (4)
C6B—C3B—H3B	109.1	C14D—C13D—C12D	118.7 (6)
C2B—C3B—H3B	109.1	C14D—C13D—H13D	120.6
C4B—C3B—H3B	109.1	C12D—C13D—H13D	120.6
C5B—C4B—C21B	113.1 (3)	C13D—C14D—C15D	122.4 (5)
C5B—C4B—C3B	101.9 (3)	C13D—C14D—H14D	118.8
C21B—C4B—C3B	116.2 (3)	C15D—C14D—H14D	118.8
C5B—C4B—H4B	108.4	C14D—C15D—C16D	122.4 (6)
C21B—C4B—H4B	108.4	C14D—C15D—H15D	118.8
C3B—C4B—H4B	108.4	C16D—C15D—H15D	118.8
C1B—C5B—C12B	107.0 (4)	C15D—C16D—C11D	115.7 (5)
C1B—C5B—C4B	112.5 (4)	C15D—C16D—H16D	122.1
C12B—C5B—C4B	140.5 (4)	C11D—C16D—H16D	122.1
O2B—C6B—O3B	122.8 (4)	C22D—C21D—C26D	118.0 (4)
O2B—C6B—C3B	125.0 (4)	C22D—C21D—C4D	122.2 (4)
O3B—C6B—C3B	112.2 (4)	C26D—C21D—C4D	119.8 (4)
O3B—C7B—H7B1	109.5	C21D—C22D—C23D	121.6 (4)
O3B—C7B—H7B2	109.5	C21D—C22D—H22D	119.2
H7B1—C7B—H7B2	109.5	C23D—C22D—H22D	119.2
O3B—C7B—H7B3	109.5	C24D—C23D—C22D	118.8 (4)
H7B1—C7B—H7B3	109.5	C24D—C23D—H23D	120.6
H7B2—C7B—H7B3	109.5	C22D—C23D—H23D	120.6
N1B—C11B—C16B	128.6 (4)	C23D—C24D—C25D	121.5 (4)
N1B—C11B—C12B	109.1 (4)	C23D—C24D—Cl1D	119.4 (4)
C16B—C11B—C12B	122.2 (4)	C25D—C24D—Cl1D	119.0 (4)
C13B—C12B—C5B	135.9 (4)	C24D—C25D—C26D	118.6 (4)
C13B—C12B—C11B	118.2 (4)	C24D—C25D—H25D	120.7
C5B—C12B—C11B	105.9 (4)	C26D—C25D—H25D	120.7
C14B—C13B—C12B	119.3 (4)	C21D—C26D—C25D	121.6 (5)
C14B—C13B—H13B	120.4	C21D—C26D—H26D	119.2
C12B—C13B—H13B	120.4	C25D—C26D—H26D	119.2
C13B—C14B—C15B	121.2 (5)		
			
C11—N1—C1—C5	−1.2 (4)	N1B—C11B—C12B—C5B	−1.1 (4)
C11—N1—C1—C2	179.8 (4)	C16B—C11B—C12B—C5B	−178.6 (4)
N1—C1—C2—O1	1.3 (7)	C5B—C12B—C13B—C14B	178.7 (5)
C5—C1—C2—O1	−177.6 (4)	C11B—C12B—C13B—C14B	−0.5 (7)
N1—C1—C2—C3	−175.7 (4)	C12B—C13B—C14B—C15B	0.7 (8)
C5—C1—C2—C3	5.3 (4)	C13B—C14B—C15B—C16B	−1.2 (8)
O1—C2—C3—C6	41.6 (5)	C14B—C15B—C16B—C11B	1.4 (7)
C1—C2—C3—C6	−141.1 (3)	N1B—C11B—C16B—C15B	−178.3 (4)
O1—C2—C3—C4	169.1 (3)	C12B—C11B—C16B—C15B	−1.3 (6)
C1—C2—C3—C4	−13.7 (4)	C5B—C4B—C21B—C26B	−85.8 (5)
C6—C3—C4—C5	143.2 (3)	C3B—C4B—C21B—C26B	156.9 (4)
C2—C3—C4—C5	15.9 (3)	C5B—C4B—C21B—C22B	89.6 (5)
C6—C3—C4—C21	−92.0 (4)	C3B—C4B—C21B—C22B	−27.7 (6)
C2—C3—C4—C21	140.7 (3)	C26B—C21B—C22B—C23B	0.4 (7)
N1—C1—C5—C12	1.6 (4)	C4B—C21B—C22B—C23B	−175.0 (4)
C2—C1—C5—C12	−179.2 (3)	C21B—C22B—C23B—C24B	−1.1 (8)
N1—C1—C5—C4	−173.6 (3)	C22B—C23B—C24B—C25B	1.5 (8)
C2—C1—C5—C4	5.6 (4)	C22B—C23B—C24B—Cl1B	−179.3 (4)
C21—C4—C5—C1	−135.4 (3)	C23B—C24B—C25B—C26B	−1.1 (8)
C3—C4—C5—C1	−13.6 (4)	Cl1B—C24B—C25B—C26B	179.7 (4)
C21—C4—C5—C12	51.8 (6)	C22B—C21B—C26B—C25B	0.0 (7)
C3—C4—C5—C12	173.5 (5)	C4B—C21B—C26B—C25B	175.6 (4)
C7—O3—C6—O2	5.9 (5)	C24B—C25B—C26B—C21B	0.3 (8)
C7—O3—C6—C3	−173.6 (3)	C11C—N1C—C1C—C5C	−0.8 (6)
C2—C3—C6—O2	109.5 (4)	C11C—N1C—C1C—C2C	176.3 (6)
C4—C3—C6—O2	−14.2 (5)	N1C—C1C—C2C—O1C	7.5 (11)
C2—C3—C6—O3	−71.1 (4)	C5C—C1C—C2C—O1C	−175.4 (6)
C4—C3—C6—O3	165.3 (3)	N1C—C1C—C2C—C3C	−170.6 (6)
C1—N1—C11—C16	−179.5 (4)	C5C—C1C—C2C—C3C	6.5 (5)
C1—N1—C11—C12	0.4 (4)	O1C—C2C—C3C—C6C	40.8 (6)
C1—C5—C12—C13	179.4 (4)	C1C—C2C—C3C—C6C	−140.9 (4)
C4—C5—C12—C13	−7.5 (8)	O1C—C2C—C3C—C4C	166.7 (5)
C1—C5—C12—C11	−1.2 (4)	C1C—C2C—C3C—C4C	−15.1 (5)
C4—C5—C12—C11	171.9 (4)	C6C—C3C—C4C—C5C	139.3 (4)
N1—C11—C12—C5	0.5 (4)	C2C—C3C—C4C—C5C	17.0 (5)
C16—C11—C12—C5	−179.6 (3)	C6C—C3C—C4C—C21C	−96.0 (5)
N1—C11—C12—C13	−180.0 (3)	C2C—C3C—C4C—C21C	141.7 (4)
C16—C11—C12—C13	0.0 (6)	N1C—C1C—C5C—C12C	−0.6 (6)
C5—C12—C13—C14	179.8 (4)	C2C—C1C—C5C—C12C	−178.5 (4)
C11—C12—C13—C14	0.3 (6)	N1C—C1C—C5C—C4C	−176.9 (4)
C12—C13—C14—C15	−0.7 (7)	C2C—C1C—C5C—C4C	5.2 (6)
C13—C14—C15—C16	0.9 (7)	C21C—C4C—C5C—C1C	−139.4 (4)
C14—C15—C16—C11	−0.5 (6)	C3C—C4C—C5C—C1C	−14.0 (5)
N1—C11—C16—C15	−180.0 (4)	C21C—C4C—C5C—C12C	46.2 (8)
C12—C11—C16—C15	0.1 (6)	C3C—C4C—C5C—C12C	171.5 (6)
C5—C4—C21—C26	−144.1 (3)	C7C—O3C—C6C—O2C	25.6 (9)
C3—C4—C21—C26	100.3 (4)	C7C—O3C—C6C—O2C'	−24.2 (7)
C5—C4—C21—C22	40.4 (5)	C7C—O3C—C6C—C3C	176.6 (4)
C3—C4—C21—C22	−75.2 (4)	C2C—C3C—C6C—O2C	64.1 (7)
C26—C21—C22—C23	−1.6 (6)	C4C—C3C—C6C—O2C	−56.3 (7)
C4—C21—C22—C23	174.0 (4)	C2C—C3C—C6C—O2C'	114.8 (7)
C21—C22—C23—C24	1.0 (6)	C4C—C3C—C6C—O2C'	−5.6 (9)
C22—C23—C24—C25	0.3 (6)	C2C—C3C—C6C—O3C	−89.6 (5)
C22—C23—C24—Cl1	−175.7 (3)	C4C—C3C—C6C—O3C	150.0 (4)
C23—C24—C25—C26	−0.8 (6)	C1C—N1C—C11C—C16C	−177.6 (5)
Cl1—C24—C25—C26	175.0 (3)	C1C—N1C—C11C—C12C	1.9 (5)
C24—C25—C26—C21	0.2 (6)	C16C—C11C—C12C—C13C	0.4 (9)
C22—C21—C26—C25	1.0 (6)	N1C—C11C—C12C—C13C	−179.1 (7)
C4—C21—C26—C25	−174.6 (4)	C16C—C11C—C12C—C5C	177.3 (5)
C11A—N1A—C1A—C5A	−1.3 (5)	N1C—C11C—C12C—C5C	−2.3 (6)
C11A—N1A—C1A—C2A	−179.6 (4)	C1C—C5C—C12C—C13C	177.7 (9)
C5A—C1A—C2A—O1A	−178.3 (4)	C4C—C5C—C12C—C13C	−7.6 (14)
N1A—C1A—C2A—O1A	0.1 (8)	C1C—C5C—C12C—C11C	1.7 (5)
C5A—C1A—C2A—C3A	2.7 (5)	C4C—C5C—C12C—C11C	176.3 (6)
N1A—C1A—C2A—C3A	−179.0 (4)	C11C—C12C—C13C—C14C	−4.6 (15)
O1A—C2A—C3A—C6A	47.0 (5)	C5C—C12C—C13C—C14C	179.7 (9)
C1A—C2A—C3A—C6A	−133.8 (3)	C12C—C13C—C14C—C15C	9(2)
O1A—C2A—C3A—C4A	171.5 (4)	C13C—C14C—C15C—C16C	−8.2 (19)
C1A—C2A—C3A—C4A	−9.3 (4)	N1C—C11C—C16C—C15C	179.4 (6)
C6A—C3A—C4A—C21A	−99.5 (4)	C12C—C11C—C16C—C15C	0.0 (8)
C2A—C3A—C4A—C21A	141.2 (3)	C14C—C15C—C16C—C11C	3.8 (12)
C6A—C3A—C4A—C5A	130.9 (3)	C5C—C4C—C21C—C22C	−129.1 (5)
C2A—C3A—C4A—C5A	11.7 (4)	C3C—C4C—C21C—C22C	113.8 (5)
N1A—C1A—C5A—C12A	1.2 (5)	C5C—C4C—C21C—C26C	49.7 (7)
C2A—C1A—C5A—C12A	180.0 (3)	C3C—C4C—C21C—C26C	−67.4 (7)
N1A—C1A—C5A—C4A	−173.3 (3)	C26C—C21C—C22C—C23C	−1.5 (8)
C2A—C1A—C5A—C4A	5.5 (5)	C4C—C21C—C22C—C23C	177.4 (5)
C21A—C4A—C5A—C1A	−135.3 (3)	C21C—C22C—C23C—C24C	3.1 (9)
C3A—C4A—C5A—C1A	−10.7 (4)	C22C—C23C—C24C—C25C	−2.5 (9)
C21A—C4A—C5A—C12A	52.9 (6)	C22C—C23C—C24C—Cl1C	178.3 (4)
C3A—C4A—C5A—C12A	177.5 (5)	C23C—C24C—C25C—C26C	0.3 (9)
C7A—O3A—C6A—O2A	−5.2 (6)	Cl1C—C24C—C25C—C26C	179.5 (5)
C7A—O3A—C6A—C3A	170.9 (4)	C22C—C21C—C26C—C25C	−0.7 (9)
C2A—C3A—C6A—O2A	87.8 (4)	C4C—C21C—C26C—C25C	−179.5 (5)
C4A—C3A—C6A—O2A	−31.4 (5)	C24C—C25C—C26C—C21C	1.3 (10)
C2A—C3A—C6A—O3A	−88.2 (4)	C11D—N1D—C1D—C5D	0.4 (5)
C4A—C3A—C6A—O3A	152.6 (3)	C11D—N1D—C1D—C2D	−175.2 (5)
C1A—N1A—C11A—C16A	−178.5 (4)	C5D—C1D—C2D—O1D	−176.8 (5)
C1A—N1A—C11A—C12A	0.8 (5)	N1D—C1D—C2D—O1D	−1.2 (8)
N1A—C11A—C12A—C13A	−179.4 (4)	C5D—C1D—C2D—C3D	5.9 (5)
C16A—C11A—C12A—C13A	−0.1 (6)	N1D—C1D—C2D—C3D	−178.5 (5)
N1A—C11A—C12A—C5A	−0.1 (4)	O1D—C2D—C3D—C6D	51.8 (6)
C16A—C11A—C12A—C5A	179.3 (4)	C1D—C2D—C3D—C6D	−130.7 (4)
C1A—C5A—C12A—C13A	178.5 (5)	O1D—C2D—C3D—C4D	174.9 (4)
C4A—C5A—C12A—C13A	−9.4 (8)	C1D—C2D—C3D—C4D	−7.6 (4)
C1A—C5A—C12A—C11A	−0.7 (4)	C6D—C3D—C4D—C5D	128.1 (4)
C4A—C5A—C12A—C11A	171.4 (4)	C2D—C3D—C4D—C5D	6.5 (4)
C11A—C12A—C13A—C14A	1.4 (7)	C6D—C3D—C4D—C21D	−107.3 (4)
C5A—C12A—C13A—C14A	−177.7 (5)	C2D—C3D—C4D—C21D	131.1 (4)
C12A—C13A—C14A—C15A	−2.8 (8)	N1D—C1D—C5D—C12D	−1.4 (5)
C13A—C14A—C15A—C16A	2.9 (10)	C2D—C1D—C5D—C12D	175.4 (4)
C14A—C15A—C16A—C11A	−1.4 (9)	N1D—C1D—C5D—C4D	−178.4 (3)
N1A—C11A—C16A—C15A	179.2 (5)	C2D—C1D—C5D—C4D	−1.6 (5)
C12A—C11A—C16A—C15A	0.0 (7)	C21D—C4D—C5D—C1D	−126.6 (4)
C5A—C4A—C21A—C26A	−0.1 (5)	C3D—C4D—C5D—C1D	−3.3 (5)
C3A—C4A—C21A—C26A	−118.9 (4)	C21D—C4D—C5D—C12D	57.9 (7)
C5A—C4A—C21A—C22A	−179.5 (3)	C3D—C4D—C5D—C12D	−178.8 (5)
C3A—C4A—C21A—C22A	61.7 (4)	C7D'—O3D'—C6D—O2D	46.5 (8)
C26A—C21A—C22A—C23A	0.8 (5)	C7D'—O3D'—C6D—O2D'	4.3 (10)
C4A—C21A—C22A—C23A	−179.8 (3)	C7D'—O3D'—C6D—C3D	179.7 (5)
C21A—C22A—C23A—C24A	−1.2 (6)	C7D'—O3D'—C6D—O3D	−78.9 (7)
C22A—C23A—C24A—C25A	0.3 (6)	C2D—C3D—C6D—O2D	16.4 (11)
C22A—C23A—C24A—Cl1A	179.4 (3)	C4D—C3D—C6D—O2D	−102.8 (10)
C23A—C24A—C25A—C26A	1.0 (6)	C2D—C3D—C6D—O3D'	−77.4 (6)
Cl1A—C24A—C25A—C26A	−178.0 (3)	C4D—C3D—C6D—O3D'	163.4 (4)
C22A—C21A—C26A—C25A	0.7 (5)	C2D—C3D—C6D—O2D'	98.2 (6)
C4A—C21A—C26A—C25A	−178.7 (3)	C4D—C3D—C6D—O2D'	−21.0 (7)
C24A—C25A—C26A—C21A	−1.5 (5)	C2D—C3D—C6D—O3D	−141.8 (5)
C11B—N1B—C1B—C5B	0.1 (5)	C4D—C3D—C6D—O3D	99.0 (6)
C11B—N1B—C1B—C2B	−178.2 (5)	C7D—O3D—C6D—O2D	16.7 (13)
C5B—C1B—C2B—O1B	−173.4 (5)	C7D—O3D—C6D—O3D'	75.1 (10)
N1B—C1B—C2B—O1B	5.0 (9)	C7D—O3D—C6D—O2D'	−48.6 (11)
C5B—C1B—C2B—C3B	4.3 (5)	C7D—O3D—C6D—C3D	−179.7 (9)
N1B—C1B—C2B—C3B	−177.4 (5)	C1D—N1D—C11D—C16D	179.7 (4)
O1B—C2B—C3B—C6B	49.4 (6)	C1D—N1D—C11D—C12D	0.8 (5)
C1B—C2B—C3B—C6B	−128.4 (4)	N1D—C11D—C12D—C13D	179.4 (5)
O1B—C2B—C3B—C4B	172.2 (4)	C16D—C11D—C12D—C13D	0.5 (8)
C1B—C2B—C3B—C4B	−5.6 (5)	N1D—C11D—C12D—C5D	−1.6 (5)
C6B—C3B—C4B—C5B	125.2 (3)	C16D—C11D—C12D—C5D	179.5 (4)
C2B—C3B—C4B—C5B	4.8 (4)	C1D—C5D—C12D—C13D	−179.5 (7)
C6B—C3B—C4B—C21B	−111.4 (4)	C4D—C5D—C12D—C13D	−3.8 (11)
C2B—C3B—C4B—C21B	128.2 (4)	C1D—C5D—C12D—C11D	1.8 (5)
N1B—C1B—C5B—C12B	−0.8 (5)	C4D—C5D—C12D—C11D	177.5 (5)
C2B—C1B—C5B—C12B	177.9 (4)	C11D—C12D—C13D—C14D	−0.9 (11)
N1B—C1B—C5B—C4B	−179.9 (3)	C5D—C12D—C13D—C14D	−179.5 (7)
C2B—C1B—C5B—C4B	−1.1 (5)	C12D—C13D—C14D—C15D	0.6 (14)
C21B—C4B—C5B—C1B	−128.0 (4)	C13D—C14D—C15D—C16D	0.1 (14)
C3B—C4B—C5B—C1B	−2.5 (4)	C14D—C15D—C16D—C11D	−0.5 (10)
C21B—C4B—C5B—C12B	53.4 (7)	N1D—C11D—C16D—C15D	−178.5 (5)
C3B—C4B—C5B—C12B	178.9 (5)	C12D—C11D—C16D—C15D	0.2 (7)
C7B—O3B—C6B—O2B	−0.4 (6)	C5D—C4D—C21D—C22D	76.6 (5)
C7B—O3B—C6B—C3B	179.7 (3)	C3D—C4D—C21D—C22D	−40.4 (6)
C2B—C3B—C6B—O2B	84.2 (5)	C5D—C4D—C21D—C26D	−100.3 (6)
C4B—C3B—C6B—O2B	−35.2 (6)	C3D—C4D—C21D—C26D	142.7 (5)
C2B—C3B—C6B—O3B	−95.9 (4)	C26D—C21D—C22D—C23D	−3.1 (7)
C4B—C3B—C6B—O3B	144.7 (3)	C4D—C21D—C22D—C23D	179.9 (4)
C1B—N1B—C11B—C16B	177.9 (4)	C21D—C22D—C23D—C24D	1.5 (7)
C1B—N1B—C11B—C12B	0.6 (5)	C22D—C23D—C24D—C25D	1.1 (7)
C1B—C5B—C12B—C13B	−178.2 (5)	C22D—C23D—C24D—Cl1D	−178.2 (3)
C4B—C5B—C12B—C13B	0.5 (9)	C23D—C24D—C25D—C26D	−1.9 (8)
C1B—C5B—C12B—C11B	1.2 (4)	Cl1D—C24D—C25D—C26D	177.4 (4)
C4B—C5B—C12B—C11B	179.8 (5)	C22D—C21D—C26D—C25D	2.3 (8)
N1B—C11B—C12B—C13B	178.4 (4)	C4D—C21D—C26D—C25D	179.3 (5)
C16B—C11B—C12B—C13B	0.9 (6)	C24D—C25D—C26D—C21D	0.1 (9)

Symmetry codes: (i) −*x*, *y*, −*z*.

### Hydrogen-bond geometry (Å, °)

**Table:**

*D*—H···*A*	*D*—H	H···*A*	*D*···*A*	*D*—H···*A*
N1—H1···O1^ii^	0.76 (5)	2.24 (5)	2.827 (4)	134 (5)
N1A—H1A···O1B	0.86 (5)	2.13 (5)	2.992 (5)	172 (4)
N1B—H1B···O1A	0.90 (6)	1.90 (6)	2.767 (5)	161 (5)
N1C—H1C···O1D	1.04 (7)	1.97 (7)	2.966 (5)	160 (6)
N1D—H1D···O1C	0.95 (5)	1.89 (5)	2.806 (5)	159 (4)

Symmetry codes: (ii) −*x*+3/2, *y*−1/2, −*z*+2.
